# Isolation and characterization of serum albumin from *Camelus dromedarius*

**DOI:** 10.3892/etm.2013.1145

**Published:** 2013-06-06

**Authors:** AJAMALUDDIN MALIK, ABDULRAHMAN AL-SENAIDY, EWA SKRZYPCZAK-JANKUN, JERZY JANKUN

**Affiliations:** 1 Protein Research Chair, Department of Biochemistry, College of Sciences, King Saud University, Riyadh 11451, Kingdom of Saudi Arabia;; 2 Department of Clinical Nutrition, Medical University of Gdańsk, 80-211 Gdańsk, Poland;; 3 Urology Research Center, Department of Urology, The University of Toledo, Health Science Campus, Toledo, OH 43614, USA

**Keywords:** camel serum albumin, protein purification and characterization, capillary electrophoresis, high-performance liquid chromatography

## Abstract

Serum albumin constitutes 35–50 mg/ml of plasma proteins and performs various physiological activities including the regulation of osmotic pressure on blood, maintaining buffering of the blood pH, carrying different fatty acids and other small molecules, such as bilirubin, hormones, drugs and metal ions, as well as participating in immunological responses. Serum albumin is an extensively used protein in biotechnological and pharmaceutical industries. The camel (*Camelus dromedarius*) is well tailored to successfully survive in extremely hot and dry climates. Plasma osmolality in the camel increases during water-deprived conditions. In such circumstances serum albumin is crucial in the regulation of blood pressure. The study of biochemical, biophysical and immunological aspects of camel serum albumin (CSA) are likely to provide molecular insights into camel physiology and may render it an alternative to human serum albumin (HSA) and bovine serum albumin (BSA) in all cases. However, these proteins are currently not available or cannot be utilized due to a variety of considerations. In this study, 12 mg of highly pure CSA was obtained from 1 ml plasma. Coomassie Brilliant Blue staining of SDS-PAGE yielded one band and RP-HPLC results revealed a single sharp peak, indicating homogenous preparation of the CSA. The charge/mass ratio and surface hydrophobicity of the CSA was similar to that of BSA. Mass spectrometry analysis of the purified protein confirmed the identity of CSA.

## Introduction

Serum albumin is synthesized mainly in the liver (1) and secreted into blood where it constitutes almost 50% of the proteins in the serum (2–5). Albumin also produces the majority of the protein contents of extracellular fluids such as interstitial, cerebrospinal, and lymphatic (2,6,7). Albumins are simple proteins without prosthetic groups, glycosylation or lipid post-translational modification. Serum albumin is a highly soluble, single-chain polypeptide comprising almost 585 amino acids (8–10). The development of the serum albumin gene has occurred due to gene triplication of monomeric serum albumin (11,12). Results of multiple sequence analysis of serum albumin revealed the well-characterized replication of three long homologous domains of 180 amino acids (13). Results of X-ray crystallographic analyses of various albumins revealed that their structures consist of only α helices (∼67%), connected by long flexible loops (14,15). The 3D structure analysis of serum albumin also demonstrated that this heart-shaped globular protein comprises three homologous domains (I, II and III), each of which consist of sub-domains A and B (16). Albumin is a disulfide-rich protein (17 disulfide bonds) that provides rigidity in the molecule while the long connecting loops provide flexibility and binding properties (17–19).

Serum albumin performs several vital functions (2,20,21) including transport of hormones, endogenous and exogenous molecules, fatty acids, bilirubin, numerous substances that are toxic in free form, as well as involvement in inflammatory responses, and prevention of the photo-degradation of folic acid (2,22–25). Serum albumins are multifunctional and economically viable due to their extensive use in the pharmaceutical and biotechnological industries (26,27). They are employed as stabilizers in therapeutic proteins, vaccines and enzymes (28–30). Serum albumins are amphiphilic in nature and could be utilized to prevent adsorption of other active proteins on the surface of the container, or could be included in the formulation to suppress protein aggregation (31,32).

The camel (*Camelus dromedarius*) is an extremely adaptable mammal capable of surviving in extremely hot climates without water intake for several weeks. Therefore, camel serum albumin (CSA) may possess unique ligand binding and stability/folding properties. In the present study, we have adopted a chromatographic method that may be used to purify CSA to homogeneity and characterized some of its properties using capillary electrophoresis, high-performance liquid chromatography (HPLC) and mass spectrometry. The purpose of this study was to explore the possibility of substituting HSA/BSA with CSA in all cases. However, these proteins are currently not available or cannot be utilized due to variety of considerations.

## Materials and methods

### Materials

Fresh camel blood was purchased from the slaughterhouse. Blue-Sepharose, Q-Sepharose and Sephacryl S-100 column, as well as low molecular weight (LMW) markers were obtained from GE Healthcare (Pittsburgh, PA, USA). ÄKTA purifier and SDS-PAGE assembly were from GE Healthcare. HPLC (Agilent 1260 Infinity LC system) and capillary electrophoresis (CE) (7100 capillary electrophoresis) were from Agilent Technologies (Santa Clara, CA, USA). Mass spectrometry-based proteomics analysis was performed at the Proteomics Resource Facility, University of Michigan, MI, USA, using multidimensional proteomic identification technology. All the chemicals used were of analytical grade.

### Plasma preparation

Camel blood was collected in a beaker containing anticoagulant (10 mg/ml EDTA). Anticoagulant was mixed gently using glass rod. Blood samples were transported on ice. Clear supernatant was separated following centrifugation at 1,000 × g for 30 min at 4°C. Plasma was aliquoted and stored at −80°C.

### Purification of CSA on Blue-Sepharose column

Frozen plasma was thawed on ice and 5 ml of plasma aliquot was centrifuged at 24,000 × g for 10 min at 4°C to remove debris. The supernatant was passed through a 0.45 micron syringe filter. To reduce ionic strength, plasma was diluted 10-fold in 20 mM Tris-HCl, pH 8.0. The Blue-Sepharose column was equilibrated with 20 mM Tris-HCl, pH 8.0. Diluted plasma was passed through pre-equilibrated Blue-Sepharose column. Flow-through was collected for further analysis. The column was washed extensively (5 CV) with 20 mM Tris-HCl, pH 8.0. Bound protein was eluted with a linear gradient of NaCl; the elution buffer was 20 mM Tris-HCl, 2 M NaCl, pH 8.0. Fractions were loaded on 12% SDS-PAGE to analyze the purity. Fractions containing a band corresponding to albumin were pooled and concentrated by Amicon 8050 stirred cells. Concentrated pooled protein was dialyzed (1:100 v/v) twice against 20 mM Tris-HCl, pH 8.0.

### Purification of CSA on Q-Sepharose

Dialyzed protein sample was centrifuged at 24,000 × g for 30 min at 4°C and filtered through 0.45 micron filter. Protein sample was passed through a pre-equilibrated (20 mM Tris-HCl, pH 8.0) Q-Sepharose column using a syringe. The column was washed with 10 CV of 20 mM Tris-HCl, pH 8.0. Bound proteins were eluted on fast protein liquid chromatography (FPLC) with a buffer of 20 mM Tris-HCl, 2 M NaCl, pH 8.0 using a 0–50% gradient of 2 M NaCl for 50 min at a 1 ml/min flow rate. Purity of the eluted fractions was analyzed by 12% SDS-PAGE and pure fractions were pooled for gel filtration chromatography.

### Purification of CSA by gel filtration

Sephacryl S-100 column was equilibrated with 20 mM Tris-HCl, 300 mM NaCl, pH 8.0. Protein sample was loaded using 10 ml superloop. Fractions were collected at a 1 ml/ml flow-rate. Purity of the fractions was analyzed by 12% SDS-PAGE. Pure fractions were pooled and dialyzed three times against water (1:100 v/v).

### Multiple sequence alignment

Partial mRNA sequence of CSA (NCBI accession no. HM640019.1) was translated into protein sequences using Ex PASy web tools (http://web.expasy.org/translate/). Multiple sequence alignment was performed using the Jalview program (http://www.jalview.org/) between human (P02768), bovine (P02769), horse (P35747), and rabbit (P49065), as well as the partial sequence of CSA.

### Mass spectrometry and protein analysis

Gel proteolysis was performed as described in a previous study (33). The protein band stained with Coomassie Brilliant Blue was excised from 12% gradient SDS-PAGE gel. The excised gel piece was destained for 4 h in 10 ml of 30% methanol. After destaining, the excised gel piece was incubated for 30 min in 200 *μ*l of 1:1 mixture of 100 mM ammonium bicarbonate buffer with acetonitrile (buffer A). The gel was then transferred into a reducing buffer comprising 10 mM dithiothreitol (DTT) in 100 mM ammonium bicarbonate buffer, and was incubated for 30 min. After washing in 150 *μ*l of buffer A, 150 *μ*l of alkylation buffer was added (50 mM iodoacetamide in 100 mM ammonium bicarbonate). After two washes in buffer A for 5 min each, the gel plugs were diced/crushed into smaller cubes followed by drying in Speed Vac for 10 min. Then, 30 *μ*l (750 ng) of trypsin solution was added to the dried gel pieces and the gel was swollen for 10 min at room temperature. Subsequently, 50 *μ*l of 100 mM ammonium bicarbonate buffer was added until the gel pieces were submerged. This was followed by incubation at 37°C for 12 h and another 10 *μ*l (250 ng) of sequencing grade trypsin was added (Promega, Madison, WI, USA) and incubated for an additional 2 h. The digest was removed and 150 *μ*l of 60% acetonitrile and 0.1% trifluroacetic acid (TFA) (buffer B) were added followed by incubation for 30 min at 30°C. The solution was removed and pooled with solution from the previous step. Extraction was repeated and solutions were concentrated to a final volume of 15–20 *μ*l.

Digest (2 *μ*l) was separated on a reverse phase column (Aquasil C18, 15 *μ*m tip × 75 *μ*m id × 5 cm Picofrit column, New Objectives, Woburn, MA, USA) using an acetonitrile/1% acetic acid gradient system (5–75% acetonitrile over 35 min followed by 95% acetonitrile wash for 5 min) at a flow rate of 250 nl/min. Trypsin-cleaved peptides were directly introduced into an ion-trap mass spectrometer equipped with a nanospray source. The mass spectrometer was set for analyzing the positive ions and obtaining a full MS scan and a collision-induced dissociation spectrum on the most abundant ion from the full MS scan (relative collision energy ∼30%). Dynamic exclusion was set to collect 3 CID spectra on the most abundant ion and then exclude the ion for 2 min. Data were searched against human and bovine databases appended with human (P02768), bovine (P02769), horse (P35747) and rabbit (P49065) albumin.

### SDS-PAGE analysis

Purity of albumin after the different steps of purification was analyzed by 12% SDS-PAGE. Protein samples (50 *μ*l) were mixed with 10 *μ*l 5X SDS-loading dye and boiled for 2 min. Boiled samples were centrifuged at 10,000 × g for 10 sec. From each sample, 10 *μ*l were loaded on SDS-PAGE.

### Purity of CSA analyzed by HPLC

The homogeneity of the purified CSA was analyzed by RP-HPLC. The mobile phase A consisted of 10% acetonitrile/90% water containing 0.01% TFA and mobile phase B was 90% acetonitrile/10% water containing 0.01% TFA. The column was 5 *μ*m, 4.6×150 mm waters symmetry C18 column and equilibrated with mobile phase A. Purified CSA was bound on the column and eluted with a linear gradient of mobile phase B.

### Purity of CSA by capillary zone electrophoresis

Polyvinyl alcohol (PVA)-coated CE capillary (75 *μ*m, 56 cm in length) was fitted into high sensitivity detection cell (Agilent Technologies). The capillary was extensively washed with 20 mM triethanolamine buffer, pH 3.6. Purified CSA (1 mg/ml) was diluted 10-fold in 20 mM triethanolamine buffer, pH 3.6. Hydrodynamic pressure (100 mb) was applied for 10 sec to inject samples in the capillary and electrophoresis was performed at 10 kV (positive polarity), 100 *μ*A current and 6 W in 20 mM triethanolamine buffer, pH 3.6 for 30 min.

## Results and Discussion

### Purification of CSA

**Figure 1.**
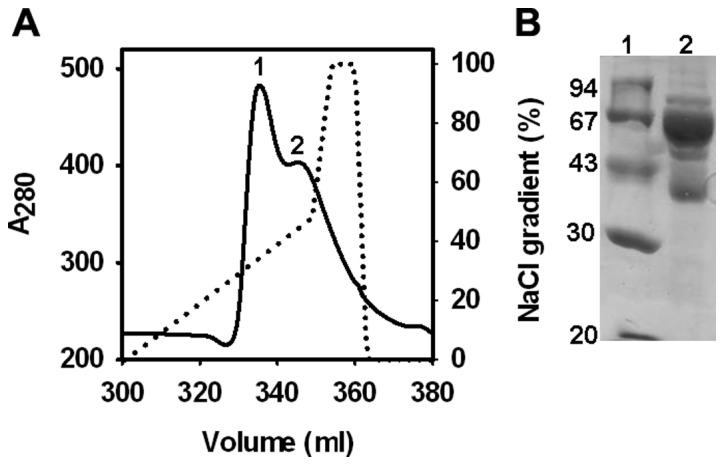
Capture of camel serum albumin (CSA) by affinity chromatography. (A) Elution profile of CSA from Cibacron Blue 3G (Blue-Sepharose). Plasma diluted 10-fold was loaded on Blue-Sepharose. The CSA bound matrix was washed with 20 mM Tris-HCl, pH 8.0. Bound protein was eluted with NaCl linear gradient (dotted line). The eluted protein is shown by the solid line. Following analysis of the purity of different eluted fractions on SDS-PAGE, peak 1 and shoulder peak 2 were pooled separately. Fractions in peak 1 were more pure than fractions in peak 2. (B) Lane 1, low molecular weight (LMW) markers; lane 2, pooled peak 1 from Blue-Sepharose was loaded on 12% SDS-PAGE.

Centrifugation of EDTA-treated camel blood at 1,000 × g resulted in slightly reddish clear plasma. When diluted plasma is passed through an equilibrated Blue-Sepharose column at a pH of 8.0, albumin binds with Cibacron Blue 3G on Blue-Sepharose 6 matrix (34). Bound protein was eluted with linear NaCl gradient (Fig. 1). It was observed that even when plasma containing 50–60 mg albumin was applied on 20 ml Blue-Sepharose column (∼360 mg HSA binding capacity), only the partial binding of CSA was observed. Cibacron Blue-Sepharose efficiently is known to eliminate almost all the albumin from human serum but not from bovine, sheep and rabbit serum (35). At pH 8.0, HSA binding on Blue-Sepharose was ∼90% while under identical conditions only ∼50% BSA binds on Blue-Sepharose (35). The *K*_diss_ of defatted human, bovine, rabbit and sheep serum albumin for Cibacron Blue were 19, 196, 150 and 150 *μ*M, respectively (36). The binding of different mammalian serum albumin on Blue-Sepharose was decreased as the degree of pre-saturation of albumins with bilirubin, fatty acids and number of carbon atoms in the chains of fatty acids increased (35,36).

**Figure 2.**
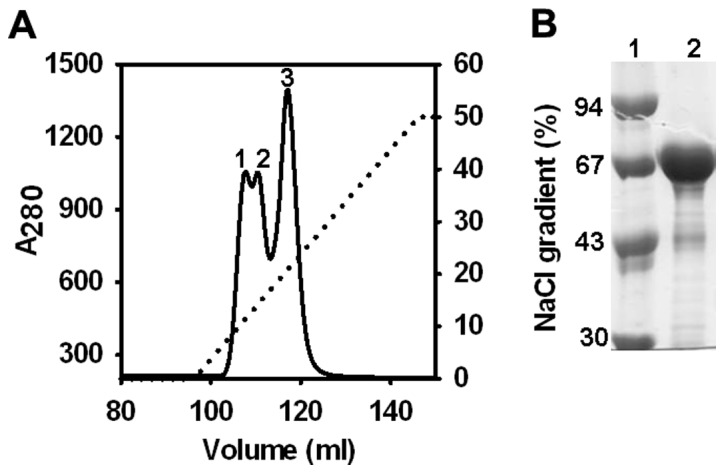
Purification of camel serum albumin (CSA) by Q-Sepharose. (A) Peak 1 eluted from Blue-Sepharose was pooled and extensively dialysed against 20 mM Tris-HCl, pH 8.0 before binding on Q-Sepharose. Washing was carried out using 20 mM Tris-HCl, pH 8.0. Bound protein was eluted with NaCl linear gradient (dotted line). Eluted protein is shown by the solid line. Following analysis of the different fractions on 12% SDS-PAGE, relatively pure fractions (present in the 3rd peak) were pooled for further purification. (B) SDS-PAGE analysis. Lane 1, low molecular weight (LMW) markers; lane 2, pooled peak 3 eluted from Q-Sepharose was loaded on 12% SDS-PAGE.

**Figure 3.**
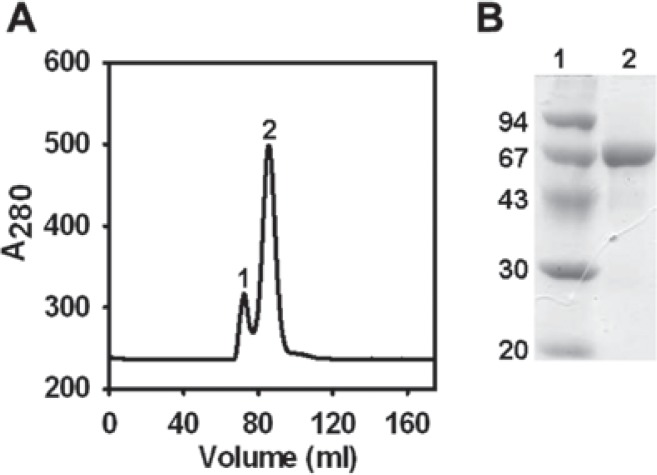
Polishing of camel serum albumin (CSA) by gel filtration chromatography. (A) Pooled 3rd peak from Q-Sepharose was loaded on Sephacryl S-100. The solid line is the eluted protein from Sephacryl S-100. Eluted fractions were analyzed on 12% SDS-PAGE for purity. (B) SDS-PAGE analysis of purified CSA. Lane 1, low molecular weight (LMW) markers; lane 2, pooled peak 2 from Sephacryl S-100.

Following enrichment of CSA by Blue-Sepharose affinity chromatography, partially purified CSA was further purified by 5 ml HiTrap Q column (250 mg HSA binding capacity). At pH 8.0, CSA was efficiently and tightly bound on HiTrap Q column. No CSA was detected in the flow through and wash. Bound albumin was eluted by the linear NaCl gradient and purity was analyzed by 12% SDS-PAGE (Fig. 2). In the polishing step of purification, gel permeation chromatography was performed using Sephacryl S-100 (Fig. 3). Using this protocol, highly pure CSA was obtained reproducibly at ∼12 mg level from 1 ml of camel plasma in three independent experiments.

### Identification of purified protein by mass spectrometry

**Figure 4.**
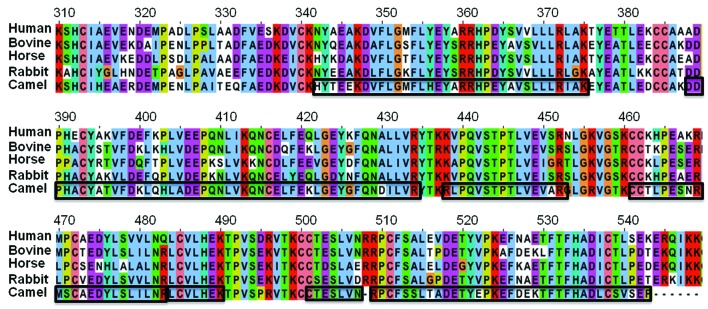
Multiple sequence alignment. Partial sequence of camel serum albumin (CSA) (NCBI accession no. HM640019.1) was aligned with human (P02768), bovine (P02769), horse (P35747) and rabbit albumin (P49065) using MAFFT multiple sequence alignment program. Residues are color coded according to conservancy. Boxed albumin sequences correspond to peptides identified by mass-spectrometry.

SDS-PAGE of purified CSA was carried out and protein band corresponding to the molecular weight of albumin (68 kDa) was excised with a clean razor. Sliced gel was placed in a sterilized Eppendorf tube and subjected to mass spectrometry. As shown in Fig. 4, multiple fragments of CSA were identified. Only the partial sequence of CSA (GenBank: HM640019.1) was deposited in the gene bank (http://getentry.ddbj.nig.ac.jp/getentry/ddbj/HM640019?filetype=html). When this partial sequence of CSA was aligned with the corresponding sequence of human, bovine, rabbit and horse, 75, 79, 71 and 69% similarities were identified, respectively. Mass spectrometric data revealed unique sequences (of 7–26 amino acids in length) that correspond to the known partial sequence of CSA (Fig. 4). These data also indicated that identified peptides overlapped with the unique sequence of CSA and confirmed that the purified protein was CSA.

### Analysis of CSA by RP-HPLC

**Figure 5.**
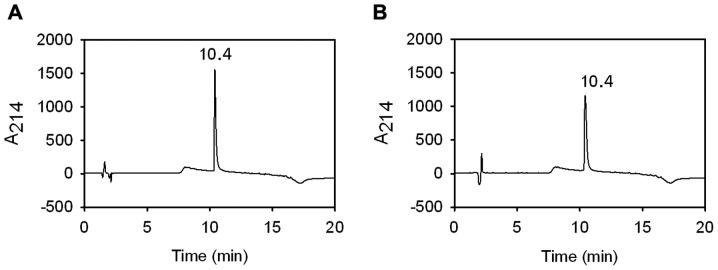
High-performance liquid chromatography (HPLC) analysis of camel serum albumin (CSA) and bovine serum albumin (BSA). Homogeneity of the purified CSA and surface hydrophobicity of the CSA was compared with commercial BSA by HPLC analysis. (A) Purified CSA and (B) commercial BSA were loaded on C18 column. Bound CSA was eluted in the single peak and corresponded to the same retention time as BSA, indicating similar surface hydrophobicity in the two albumins.

The purity and homogeneity of the purified proteins was analyzed by RP-HPLC, which separate the proteins based upon surface hydrophobicity. To separate proteins, acetonitrile was frequently used as an organic modifier and TFA as the ion-pairing agent (37). When a linear gradient of mobile phase B at room temperature was applied, CSA was eluted in a single and sharp peak (Fig. 5). The peak retention time for purified CSA was 10.4 min, which was the same as for commercial BSA.

### Analysis of CSA by capillary zone electrophoresis

**Figure 6.**
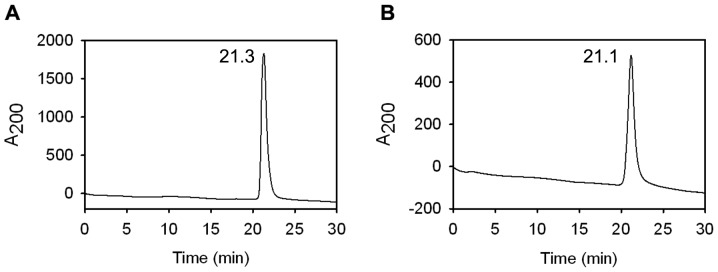
Capillary zone electrophoresis analysis of camel serum albumin (CSA) and bovine serum albumin (BSA). Charge to mass ratio of CSA was compared with BSA in capillary zone electrophoresis mode. (A) Purified CSA and (B) commercial BSA was passed through polyvinyl alcohol (PVA)-coated capillary at pH 3.6. Purified CSA was eluted in a single symmetrical peak and the retention time of CSA and BSA were similar, indicating a similar charge/mass ratio.

Capillary zone electrophoresis (CZE) separates proteins based on their unique charge/mass ratio. CZE is a complementary technique to RP-HPLC used for determination of the homogeneity of proteins (38). The capillary zone electropherogram of purified CSA showed a single and symmetrical peak (Fig. 6A), which confirmed the homogenous preparation of CSA. The retention time of CSA and commercial BSA (Fig. 6B) were very close (21.3 vs. 21.1 min).

### Conclusion

Findings of SDS-PAGE, RP-HPLC and CZE indicate that the current protocol for the isolation of CSA is efficient, with a high yield and of pure grade. The mass spectrometric data confirmed that purified protein is CSA. The described protocol for purification of CSA is simple, efficient, and reproducible. Furthermore, it yields a homogeneous product of CSA. In addition, in this procedure mild conditions were applied to minimize the denaturation of the CSA. In a number of biotechnological and routine biochemical applications, HSA is replaced with BSA (39). However, an investigation into the structural, biochemical and immunological aspects of CSA, potentially render it a viable alternative to HSA for certain applications, such as cell culture, or indicate that it may possess unique properties that may be beneficial in engineering albumin to improve various biological and physical properties including ligand binding specificity, affinity, and stability.

## Abbreviations:

LMW: low molecular weight;
PVA: polyvinyl alcohol;
CE: capillary electrophoresis;
FPLC: fast protein liquid chromatography;
CV: column volume;
TFA: trifluroacetic acid
